# Nurse-led rapid rehabilitation following mechanical thrombectomy in patients with acute ischemic stroke: A historical control study

**DOI:** 10.1097/MD.0000000000034232

**Published:** 2023-07-14

**Authors:** Yueyue He, Rui Wang, Shuju Dong, Shiyan Long, Ping Zhang, Ling Feng

**Affiliations:** a Department of Neurology, West China Hospital, Sichuan University/ West China School of Nursing, Sichuan University, Chengdu, People’s Republic of China; b Department of Neurology, West China Hospital, Sichuan University, Chengdu, People’s Republic of China.

**Keywords:** acute ischemic stroke, historical control study, mechanical thrombectomy, quality of life, rapid rehabilitation

## Abstract

The care model composed of a multidisciplinary team is the best model to promote stroke rehabilitation. The objective of this study was to explore the effect of nurse-led rapid rehabilitation on mechanical thrombectomy (MT) in patients with acute ischemic stroke (AIS). This study used a non-randomized, historically controlled clinical trial design to compare the efficacy of nurse-led rapid and routine rehabilitation after thrombectomy in patients with ischemic stroke. Treatment outcomes, including hospitalization duration, hospitalization costs, scores on multiple scales at discharge, and clinical outcomes 3 months post-discharge, were assessed and compared between the 2 rehabilitation approaches. Our report is based on the STROBE guidelines. The differences in length of stay in hospital (*P* = .018), hospitalization expense (*P* < .001), National Institutes of Health Stroke Scale score (NIHSS) score at discharge (*P* < .001), modified Rankin scale (MRS) score at discharge (*P* < .001), and ADL (Activities of Daily Living) score at discharge (*P* = .156) between the intervention group and the control group were statistically significant. There were statistically significant differences in anxiety/depression (*P* = .013) and overall quality of life (*P* = .017) 3 months after discharge. Generalized estimating equation (GEE) analysis showed that interaction effects between group and time were statistically significant for MRS (OR = 0.231, *95%* confidence interval: 0.128–0.417, *P* < .001). The hospitalization time of patients in the intervention group was shortened, and the hospitalization cost was reduced. There were differences in psychological status, and the overall quality of life and improvement of disability status was better.

## 1. Introduction

Stroke ranks as the leading cause of death and disability in Chinese people.^[1]^ Acute ischemic stroke (AIS) accounts for 60% to 80% of all strokes.^[2]^ Numerous studies have indicated that mechanical thrombectomy (MT) is a viable and safe treatment modality for enhancing patient prognosis.^[3,4]^ Nevertheless, patients may experience a range of complications, including hemiplegia, motor disorders, and neurological dysfunction, among others. These complications can significantly diminish their quality of life.^[5,6]^ Extensive research has been conducted on the “Green Channel” approach for AIS, which involves streamlining the process from symptom onset to hospital admission. This approach has reached a relatively advanced stage, reducing the interval between symptom onset and surgical intervention. The implementation of this approach has been proven to significantly contribute to enhancing patient prognoses.^[7]^ Furthermore, optimizing the rehabilitation process following MT is paramount. Numerous studies have provided compelling evidence that rehabilitation training improves patient prognoses, enhances their quality of life, and reduces mortality rates.^[8]^ Rehabilitation training methods encompass a wide range of approaches, including but not limited to active or passive training, psychological rehabilitation, occupational therapy, cognitive rehabilitation, and cardiac rehabilitation.^[9,10]^ The care model composed of a multidisciplinary team is the best model to promote stroke rehabilitation.^[11]^ However, the hindrances to early rehabilitation treatment include a shortage of rehabilitation therapists, undefined nursing roles, and unclear nursing responsibilities in the rehabilitation process.^[12]^ As long-term and continuous patient caregivers, nurses possess distinct advantages in facilitating early rehabilitation interventions.^[13]^ Thus, it is crucial to develop stroke rehabilitation nursing programs that can guide clinical nursing practices, foster the advancement of specialized stroke nursing, promote interdisciplinary collaboration, and offer optimal rehabilitation approaches.^[14]^ Recovering the ability of daily living activities is patients’ most practical rehabilitation goal but also one of the reasons to promote patients to rehabilitation.^[15]^ Assessing functional abilities using the Modified Rankin Scale (MRS) is a foundation for patient treatment, nursing, and rehabilitation. It is considered one of the most critical indicators for evaluating the effectiveness of rehabilitation interventions.^[16]^ Nurse-led rapid rehabilitation has been widely used in surgical patients, which can reduce the hospitalization cost of patients and improve the long-term quality of life and daily living activities.^[17,18]^ This study developed a multidisciplinary rapid rehabilitation nursing program for patients with AIS after MT, focusing on exploring its impact on patients’ outcomes, as detailed in the subsequent report.

## 2. Methods

### 2.1. Study design

This study used a non-randomized, pre- and post-historical controlled clinical trial design to compare the efficacy of rapid and routine rehabilitation after thrombectomy in patients with ischemic stroke. Patients hospitalized from January 1 to December 31 2020, were included in the control group for regular rehabilitation care. During this period, nurses do not play a dominant role in the rehabilitation of patients, and rehabilitation physicians develop rehabilitation plans to complete the rehabilitation of patients with AIS during the recovery period. It mainly includes movement training, active or passive limb training, pulmonary rehabilitation training, etc. There are few types and single forms of rehabilitation. And patients hospitalized from January 1 to December 31, 2021, were included in the intervention group for rapid rehabilitation. The length of hospital stay, hospital cost, quality of life, mRS, and daily living ability of patients in different schemes were compared. Treatment outcomes were compared, such as length of stay and cost of hospitalization, scores on various scales at discharge, and clinical results 3 months after discharge. Given the nature of the intervention, intervention nurses and study participants could not be blinded. The 2 groups of patients received the same social support from the hospital, government, family, friends, etc.

### 2.2. Target population

#### 2.2.1. Inclusion and exclusion criteria

The inclusion criteria were as follows: AIS patients who met the relevant requirements for MT surgery during hospitalization and were selected according to AHA/ASA 2018 AIS Early Management Guidelines; over 18 years of age; signed informed consent for vascular interventional therapy by themselves or their representatives; and first episode of AIS; Informed consent was obtained from patients or family members.

The exclusion criteria were as follows: severe heart disease, lung disease, liver disease, kidney disease, and other organ dysfunction diseases or malignant tumors; lost contact or failure to complete follow-up visits; Patients who give up treatment due to severe condition after thrombectomy.

### 2.3. Implementation plan of rapid rehabilitation

#### 2.3.1. Build multidisciplinary teams.

A multidisciplinary rehabilitation management team was established through group discussion, including nurses, neurologists, rehabilitation physicians, respiratory therapists, rehabilitation therapists, occupational therapists, speech therapists, and dietitians. After the formation of the protocol and before the formal implementation of the study, we developed a training plan for team members, and the department managers formulated the corresponding training and assessment content according to different rehabilitation duties. The teaching method was mainly a combination of online teaching and special lectures in the Department of Neurology. We were including but not limited to nurses. They were trained for 3 months for 24 hours from October 1, 2020 to December 31, 2020, and completed and passed the examination within the deadline.

#### 2.3.2. Responsibilities of team members.

The nurse responsibility is to lead the whole process of rapid rehabilitation of patients during hospitalization, carry out targeted pain care, posture management, respiratory management, personalized health education, emotional, rollover, assessment and training of swallowing function, and pat the patient on the back and sleep management, and most importantly, patient assessment and data collection of outcome indicators. Neurologists mainly diagnose and treat patients’ overall disease and complications. Before the implementation of rehabilitation, the rehabilitation protocol was reconsulted with the patient neurologist physician to ensure that the protocol did not present safety concerns for the patient. Rehabilitation physicians, respiratory therapists, rehabilitation therapists, occupational therapists, and speech therapists are responsible for developing rehabilitation programs and implementing physical therapy, including occupational therapy, staged exercise rehabilitation training, respiratory exercise, and speech therapy. Each thrombectomy patient implemented a multidisciplinary rehabilitation diagnosis and treatment program, with members performing their respective duties to jointly promote the patient recovery.

#### 2.3.3. Rapid recovery implementation process.

The nurses started the corresponding early rehabilitation program when the patient entered the ward. Through process optimization, all links are integrated and sorted, and the fast recovery standard and process of the whole process from postoperative to rehabilitation are established. When the patient is ready to be sent to the ward after the operation, the ward nurse needs to know the basic information about the patient in the operating room in advance, including the patient vital signs, whether tracheal intubation is performed, the operation method, and the operation site. The doctor decides whether to place the ventilator according to the basic situation, and the ward prepares the corresponding supplies for the patient early recovery.

A: Routine nursing after MT

It mainly includes airway and nutrition management, close monitoring of vital signs, observation of consciousness, pupils, and muscle strength, close observation of blood pressure and control of blood pressure, improvement of postoperative review results, and observation of puncture sites and arteria dorsalis pedis.

B: Rehabilitation training
  (1) Swallowing function training

It is mainly guided by rehabilitation physicians and assisted by nurses. It includes explicitly swallowing organ training, mouth opening movement, lip movement, tongue movement, breathing training, sensory training, swallowing posture training, and eating training.

(2) Position training

Nurses step by step into position training for patients with AIS, including good limb position training, semirecumbent position training, position adaptability training, and autonomous position training.

(3) Pulmonary rehabilitation

It mainly includes breathing training, cough training, lung recruitment training, aerosol inhalation of diluted sputum, and chest percussion to promote sputum discharge.

(4) Physicotherapeutics

The rehabilitation physician and neurologist assess the patient condition and specify a specific physical therapy program administered by the nurse and the rehabilitation physician, including but not limited to active and passive limb joint movements, individual recognized training, acupuncture, and moxibustion therapy, neuromuscular electrical stimulation, tension and grip training, ankle pumping exercises, and bedside activities such as left and standing. After meeting the discharge requirements, patients can be discharged from the hospital or choose more professional rehabilitation institutions for professional rehabilitation treatment.

(5) Psychological rehabilitation

In the timely assessment of awake patients, there are related psychological problems, and they immediately seek expert consultations. Nurses also should comfort patients in their daily work and give patients confidence in treatment.

### 2.4. Data collection

The primary outcome was disability status, measured by the MRS. The prognosis was the primary endpoint measure in this study and was evaluated using MRS scores.^[19]^ 0 = No symptoms at all; 1 = No significant cs; able to carry out all usual duties and activities; 2 = Slight disability; unable to carry out all previous activities but able to look after own affairs without assistance; 3 = Moderate disability; requiring some help but able to walk without assistance; 4 = Moderately severe disability; unable to walk without assistance and unable to attend to own bodily needs without assistance; 5 = Severe disability; bedridden, incontinent and requiring constant nursing care and attention; 6 = Dead. Patients were divided into a without disability to slight disability group (mRS ≤ 2) and a moderately disabled or above group (mRS ≥ 3) according to the mRS score.^[20]^

The following secondary outcomes were assessed:

The nurse assessed the activities of daily living and MRS scores at discharge and 3 months after discharge. The Modified Barthel Index (MBI) is the most commonly used scale to evaluate the ability of daily living activities globally.^[21]^ It is simple, reliable, and sensitive. The MBI total score ranges from 0 to 100, and a higher MBI score reflects a more remarkable ability to function independently.

When assessed 3 months after discharge, nurses also evaluated the patient quality of life using parts EQ-5D (Five Questions of Euro Qol 5D Quality of Life Self-esteem Questionnaire).^[22]^ The EQ-5D-5 L consists of 5 items, each covering a health-related quality of life (HRQoL) domain, namely mobility, self-care, daily activities (e.g., work, study, housework, family or leisure activities), pain or discomfort, and anxiety or depression; each item is scored on a 5-point scale: “no problems,” “slight problems,” “moderate problems,” “severe problems,” and “extreme problems/unable.” The item scores were converted into a total value score using the EQ-5D crosswalk index value calculator, in which a perfect health score is valued as a score of 100. The EQ-5D has shown validity and reliability in stroke populations and is often used in cost-effectiveness analyses.

In addition, we collected several potential covariates. The clinical data of patients were based on the clinical medical record system. We collected the general characteristics of all AIS patients after MT in our hospital from January 2020 to December 2021. It mainly includes demographic data complicated with chronic disease. Preoperative intravenous thrombolysis was performed with alteplase (rt-PA). The National Institutes of Health Stroke Scale score (NIHSS)^[23]^ scores were assessed by neurologists at admission. All NIHSS scores were determined or supervised by NIHSS-certified physicians of the neurology service. The NIHSS scale scores the severity of stroke numerically (mild, <4; moderate, <16; severe, <25; very severe, ≥25). It should be applied at the beginning and during the evolution of the stroke.^[24]^ There are specific templates to record nursing assessment findings. Nursing assessments are made at admission, discharge, and changes in condition. Data extraction was performed after 1 of the professionals involved in the study was trained to extract electronic medical record data. On admission, nurses and doctors conduct a thorough assessment and keep medical records, which can be simultaneously extracted when extracting comprehensive data. The reliability, validity, and scoring of all instruments used in this study can be found in the published protocol.

### 2.5. Statistical methods

Continuous variables are expressed as the means and standard deviations or medians with the 25th and 75th percentiles. The compromise between the 2 groups was compared by *t* test or the Wilcoxon rank sum test for continuous variables. The nonparametric statistical method was used for variance, frequencies, and percentages expressed categorical variables, and the chi-square test was used. The effects of the intervention on the study outcomes were assessed using generalized estimating equation (GEE) models. The GEE model allows for additional model specifications and accounts for the correlation of repeated measurements.^[25]^

The GEE model was used to compare the differential changes in each outcome (MRS) at the time point 3 months after discharge (T2) to the time point of discharge (T1) between groups, with adjustment for the covariates identified as described above. The time and group interaction term (Group*Time) was used to measure the differential change in each variable across time points between the groups. All tests were 2-sided; results with *P* values below .05 were considered statistically significant. All statistical analyses were performed using SPSS v.26.

### 2.6. Ethical considerations

The hospital ethics committee approved this study. This study was approved by the West China Hospital, Sichuan University ethics committee, number 2019 (728). All participants were thoroughly informed about the voluntary nature of their participation and their right to withdraw from the study at any time. Data collection was only performed after obtaining the participants’ written informed consent. This study is a hospital nursing quality improvement and non-randomized controlled study, so it is not registered. We carefully considered the study questionnaire design, collection, and data analysis stages to protect participants’ privacy. First, we only collected essential basic information anonymously, and no identified personal privacy data was recorded in this study. Second, all researchers were informed and agreed to abide by the confidentiality of the study, and these data would be only used for research analysis.

## 3. Results

### 3.1. Description of the sample

**Figure 1.**
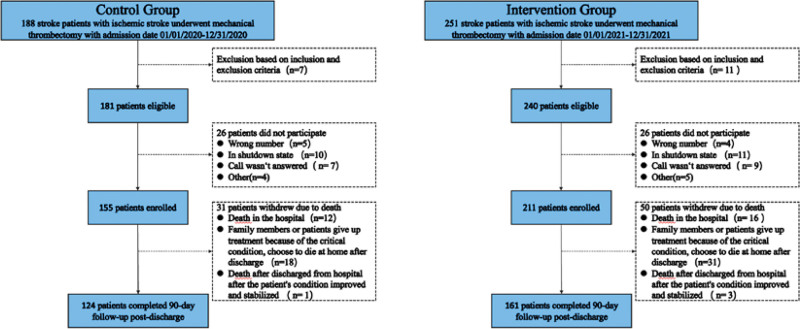
Shows the patient flow chart. It shows how the patients in our study who were finally included were determined.

For the intervention group, 251 patients with ischemic stroke who underwent MT with admission dates 01/01/2021 to 12/31/2021 were identified as potentially eligible, of which 11 were subsequently classified as unsuitable. Among the 240 eligible patients, 211 enrolled, and 50 participants withdrew due to death, resulting in 161 participants who completed the study. For the control group, 251 patients with ischemic stroke who underwent MT with admission date 01/01/2020 to 12/31/2020 were identified as potentially eligible, of which 26 were subsequently classified as unsuitable. Among the 181 eligible patients, 155 enrolled, and then 31 participants withdrew due to death, resulting in 124 participants who completed the study. This information is visually presented in Figure 1. For patients discharged from the hospital after recovery, the mortality rate was 0.806% in the control group and 1.422% in the intervention group.

124 and 161 patients were in the intervention and control groups, respectively. The characteristics of the patients in each study group at the time of admission are shown in Table 1.

**Table 1:** Demographic and clinical characteristics of patients, N = 285.

Characteristics		Control	Intervention	*P* value
		N = 155	N = 211	
Age (yr)	Mean (SD)	69.28 (13.72)	65.14 (15.45)	.017**
Gender				.379*
	Female, n (%)	51 (41.1)	58 (36.0)	
Educational level				.165*
	Illiteracy, n (%)	8 (6.5)	9 (5.6)	
	Grade school, n (%)	38 (30.6)	45 (28.0)	
	Junior middle school, n (%)	31 (25)	45 (28.0)	
	Senior high school or technical secondary school, n (%)	11 (8.9)	23 (14.3)	
	Junior college, n (%)	12 (9.7)	23 (14.3)	
	Bachelor or above, n (%)	24 (19.4)	16 (9.9)	
Marital status				.839*
	Single, n (%)	3 (2.4)	3 (1.9)	
	Married/cohabitant, n (%)	103 (83.1)	140 (87.0)	
	Widow/Widower, n (%)	16 (12.9)	16 (9.9)	
	Separated/divorced, n (%)	2 (1.6)	2 (1.2)	
Comorbidities at admission				
	Hypertension, n (%)	78 (62.9)	96 (59.6)	.574*
	Diabetes Mellitus, n (%)	31 (25.0)	43 (26.7)	.744*
	Hyperlipidemia, n (%)	29 (23.4)	37 (23.0)	.936*
	Heart Disease, n (%)	72 (58.1)	88 (54.7)	.566*
Preoperative intravenous thrombolysis was performed with alteplase (rt-PA)				.615*
	Yes, n (%)	21 (16.9)	31 (19.3)	
NIHSS score at admission	Mean (SD)	15.06 (5.71)	13.27 (7.46)	.027**

SD = standard deviation.

* P value for independent samples t test.

** P value for χ^2^-test unless otherwise specified.

### 3.2. Comparison of outcomes between the 2 groups of stroke survivors

#### 3.2.1. Outcomes during hospitalization.

The length of hospital stay, hospitalization expense, mRS score, ADL score, and NIHSS score were compared between the 2 groups. Table 2 presents the different analysis results for the study between the control and intervention groups. The differences in length of stay in the hospital (*P* = .018), hospitalization expense (*P* < .001), NIHSS score at discharge (*P* < .001), MRS score at discharge (*P* < .001), and ADL score at discharge (*P* = .156) between the intervention group and the control group were statistically significant.

**Table 2:** The treatment outcomes of both groups (N = 285).

Characteristics	Control	Intervention	*P* value
	N = 124	N = 161	
length of stay in hospital (d), M(SD)	19.35 (16.55)	15.37 (11.71)	.018
Hospitalization expense (RMB), M(SD)	139466.01 (57100.82)	114396.96 (49183.12)	<.001
NIHSS score at discharge, M(SD)	10.15 (5.69)	8.01 (5.74)	<.001
MRS score at discharge, M(SD)	2.52 (1.89)	3.61 (1.47)	<.001
ADL score at discharge, MED (IQR)	35 [IQR, 15–55]	45 [IQR, 20–60]	.156

IQR = interquartile range, M = mean, MED = median, SD = standard deviation.

#### 3.2.2. Outcomes of the 2 groups 3 months after discharge.

There was no significant difference in MRS score (*P* = .097) or ADL score (*P* = .374) between the 2 groups 3 months after discharge. There were no statistically significant differences in mobility (*P* = .349), self-ability (*P* = .394), usual activities (*P* = .897), or pain/discomfort (*P* = .383), but there were statistically significant differences in anxiety/depression (*P* = .013) and overall quality of life (*P* = .017). Table 3 presents the results of further analysis of the study between the control and intervention groups.

**Table 3:** The treatment outcomes of both groups (N = 285).

	Characteristics	Control*	Intervention*	*P* value
		N = 124	N = 161	
MRS score 3 mo after discharge		2.38 ww(1.77)	2.01 (1.95)	.097
ADL score 3 mo after discharge		71.94 (30.38)	75.19 (30.77)	.374
EQ-5D-5 L				
	Mobility	1.69 (0.78)	1.6 (0.82)	.349
	Self-care	1.56 (0.78)	1.65 (0.82)	.394
	Usual activities	1.6 (0.76)	1.61 (0.78)	.897
	Pain/discomfort	1.27 (0.48)	1.33 (0.58)	.383
	Anxiety/depression	1.39 (0.56)	1.23 (0.43)	.013
	Visual analog scale	64.72 (25.93)	72.29 (26.94)	.017

### 3.3. Effects of the Intervention on MRS

Table 2 shows that MRS Was higher in the intervention group at discharge (*P* < .001), and Table 3 shows that there was no significant difference between the 2 groups (*P* = .097). To further explore the change of MRS, this study uses the GEE model for analysis. The intervention effects on MRS are presented in Table 4. GEE analysis showed that interaction effects between group and time were statistically significant for MRS (OR = 0.231, 95% CI: 0.128–0.417, *P* < .001). From T1 to T2, a more significant decrease in the occurrence of MRS was observed in the intervention group compared with that in the control group.

**Table 4:** Generalized estimating equation model (N = 285).

	b	SE	Wald χ^2^	OR	(95%CI) Lower	(95%CI) Upper	*P*
(Intercept)	2.516	0.1693	2.184	12.381	8.884	17.254	<.001
T2	−0.137	0.2321	−0.592	0.872	0.553	1.374	.555
T1	0^a^						
Intervention group	1.093	0.205	0.691	2.982	1.995	4.457	<.001
control group	0^a^						
T2 * intervention group	−1.465	0.3012	−2.056	0.231	0.128	0.417	<.001

a This parameter is set to zero because it is redundant.

## 4. Discussion

Our study observed that stroke patients’ mortality rates within 3 months following thrombectomy were 20.0% in 2020 and 23.7% in 2021. However, the difference between the 2 years was not statistically significant. Similarly, the in-hospital mortality rates were 19.4% and 22.3%.^[26]^ Li analysis of China multicenter prospective registry showed that the mortality rate of patients with AIS treated with MT was 21.9%.^[27]^ Studies have revealed that for AIS patients with significant intracranial vessel occlusion, the advancement of endovascular interventional therapy in recent years has led to a considerably higher vascular recanalization rate with MT than intravenous thrombolysis.^[28]^

Nonetheless, the clinical outcomes of these patients have not shown substantial improvement, and they continue to face high perioperative complications and mortality rates.^[29]^ Regarding patients discharged from the hospital after a successful recovery, we observed a mortality rate of 0.806% in the control group and 1.422% in the intervention group. Despite these relatively low rates, the prognosis of AIS patients following MT in China still necessitates the attention of medical professionals. There is a crucial need for further advancements in medical technology and management strategies to enhance patient outcomes.

Better rehabilitation training is essential in improving ischemic stroke patients.^[30]^ In the rehabilitation treatment of patients, guiding patients to carry out active and passive rehabilitation training, early rehabilitation training can make patients recover and rebuild motor function as soon as possible, preventing muscle atrophy.^[31]^ In this study, compared with the control group, the hospitalization time of patients in the intervention group was shortened, and the hospitalization cost was reduced. The reason for the analysis was that the accelerated recovery nursing model was adopted after the implementation of this study, which optimized the entire rehabilitation process of ischemic stroke patients from thrombectomy to discharge. Relying on the stroke unit, multidisciplinary doctors, nurses, and therapists participate and are professionally trained to integrate first aid, treatment, nursing, and stroke rehabilitation. The standardized diagnosis, treatment, and rehabilitation of patients with AIS after MT can effectively shorten the length of hospital stay, reduce the cost of hospitalization, and create conditions for the full rehabilitation of the patients.

Furthermore, the intervention group patients were discharged when the MRS score was higher than that of the control group, but the NIHSS score was lower than that of the control group. Here, we can see that we are labeling patients who can be discharged when the choice, mainly on the recovery of neurological function, is consistent with the idea of rapid recovery. After neural function in patients with basic replies, they can continue to rest after discharge or choose more professional rehabilitation hospitals. Corresponding neurological function exercises can improve the prognosis of patients.

This study results suggest no difference in disability status and self-care ability after 3 months but that psychological status and overall quality of life were better. The better psychological condition may be related to implementing psychological rehabilitation during hospitalization. Patients can receive better psychological support through professional psychological guidance, consistent with Lee research results.^[32]^ After meeting the discharge requirements, patients could be discharged or choose more professional rehabilitation institutions for professional rehabilitation treatment. Therefore, the disability status of patients in the intervention group at discharge was not better than that of the control group. Still, the improvement in disability status 3 months after discharge was significantly better than that of the control group. To optimize medical resources, we discharged patients when their neurological function had recovered enough to qualify for discharge, but there was still a recovery period from disability. We found that the reduction of MRS. The intervention group was greater than that in the control group after 3 months, which suggested that the disability function of the patients had been improved to a greater extent through the implementation of fast-track rehabilitation in this study. These findings are consistent with numerous previous studies,^[33,34]^ highlighting that establishing a rapid rehabilitation pathway can effectively enhance the long-term prognosis of patients. Specifically, it contributes to improving the disability status of patients and enhancing their overall quality of life.

In conclusion, the rapid rehabilitation model for patients with AIS after MT has a significant effect and is worthy of reference and clinical promotion.

## 5. Limitations

This study was historically controlled and non-randomized. Although the difference in baseline data was not statistically significant, the influence of individual differences and medical progress cannot be eliminated due to the limitation of the study type. This study was single-center, and patients’ subjective feelings were not evaluated. In the future, expanding the sample size and synthesizing more effective rehabilitation methods are suggested to optimize the rehabilitation nursing program further.

## Acknowledgments

Thanks to all medical staff of the Department of Neurology, West China Hospital, Sichuan University, for supporting this study. LF, the corresponding author, is the head nurse in the Department of Neurology. She has been in stroke nursing for over 20 years and has rich experience in stroke nursing management.

## Author contributions

**Conceptualization:** Shuju Dong, Ling Feng.

**Data curation:** Yueyue He, Rui Wang, Shuju Dong, Shiyan Long, Ping Zhang, Ling Feng.

**Formal analysis:** Yueyue He, Rui Wang, Shuju Dong, Ping Zhang, Ling Feng.

**Funding acquisition:** Ling Feng.

**Investigation:** Yueyue He, Rui Wang, Ping Zhang, Ling Feng.

**Methodology:** Rui Wang, Shiyan Long, Ling Feng.

**Project administration:** Shiyan Long, Ping Zhang, Ling Feng.

**Resources:** Yueyue He, Shiyan Long, Ping Zhang, Ling Feng.

**Software:** Yueyue He, Rui Wang, Shuju Dong, Ping Zhang, Ling Feng.

**Supervision:** Yueyue He, Rui Wang, Shuju Dong, Ping Zhang, Ling Feng.

**Validation:** Yueyue He, Shuju Dong, Shiyan Long, Ping Zhang, Ling Feng.

**Visualization:** Shuju Dong, Ling Feng.

**Writing – original draft:** Yueyue He, Rui Wang, Ling Feng.

**Writing – review & editing:** Yueyue He, Rui Wang, Shuju Dong, Ping Zhang, Ling Feng.
